# 1,2,4-Triazolo[1,5-*a*]pyrimidines as a Novel Class of Inhibitors of the HIV-1 Reverse Transcriptase-Associated Ribonuclease H Activity

**DOI:** 10.3390/molecules25051183

**Published:** 2020-03-05

**Authors:** Jenny Desantis, Serena Massari, Angela Corona, Andrea Astolfi, Stefano Sabatini, Giuseppe Manfroni, Deborah Palazzotti, Violetta Cecchetti, Christophe Pannecouque, Enzo Tramontano, Oriana Tabarrini

**Affiliations:** 1 Department of Pharmaceutical Sciences, University of Perugia, 06123 Perugia, Italy; jenny.desantis@hotmail.it (J.D.); serena.massari@unipg.it (S.M.); andrea.astolfi@unipg.it (A.A.); stefano.sabatini@unipg.it (S.S.); giuseppe.manfroni@unipg.it (G.M.); deborah.palazzotti@studenti.unipg.it (D.P.); violetta.cecchetti@unipg.it (V.C.); 2 Department of Chemistry, Biology, and Biotechnology, University of Perugia, 06123 Perugia, Italy; 3 Department of Life and Environmental Sciences, University of Cagliari, Cittadella Universitaria di Monserrato, Monserrato, 09042 Cagliari, Italy; angela.corona@unica.it (A.C.); tramon@unica.it (E.T.); 4 Rega Institute for Medical Research, Laboratory of Virology and Chemotherapy, 3000 K.U. Leuven, Belgium; christophe.pannecouque@rega.kuleuven.be

**Keywords:** HIV-1, RNase H, 1,2,4-triazolo[1,5-*a*]pyrimidine, allosteric inhibitors, RT, AIDS

## Abstract

Despite great efforts have been made in the prevention and therapy of human immunodeficiency virus (HIV-1) infection, however the difficulty to eradicate latent viral reservoirs together with the emergence of multi-drug-resistant strains require the search for innovative agents, possibly exploiting novel mechanisms of action. In this context, the HIV-1 reverse transcriptase (RT)-associated ribonuclease H (RNase H), which is one of the few HIV-1 encoded enzymatic function still not targeted by any current drug, can be considered as an appealing target. In this work, we repurposed in-house anti-influenza derivatives based on the 1,2,4-triazolo[1,5-*a*]-pyrimidine (TZP) scaffold for their ability to inhibit HIV-1 RNase H function. Based on the results, a successive multi-step structural exploration around the TZP core was performed leading to identify catechol derivatives that inhibited RNase H in the low micromolar range without showing RT-associated polymerase inhibitory activity. The antiviral evaluation of the compounds in the MT4 cells showed any activity against HIV-1 (III_B_ strain). Molecular modelling and mutagenesis analysis suggested key interactions with an unexplored allosteric site providing insights for the future optimization of this class of RNase H inhibitors.

## 1. Introduction

Since the isolation and identification of the human immunodeficiency virus (HIV-1) as the cause of AIDS, extensive research work has been carried out that has led to 28 anti-HIV-1 drugs blocking different steps and targets within the HIV-1 replicative cycle. Their use as combination antiretroviral therapy (cART) permitted to achieve an impressive progress in the treatment and management of HIV-1 infection, thus revolutionizing its consideration from a death sentence to a chronic but controllable illness [1]. However, several limits, including side effects and the emergence of mutants resistant to more than one class of molecules can result on treatment failure [2,3], and the need for a life-long treatment often compromises the benefits of such cocktails of drugs [4]. Most importantly, a complete eradication of HIV-1 infection is still a challenge [5]. Thus, innovative anti-HIV-1 agents based on alternative mechanisms of action or exploiting new binding sites on traditional targets are still strongly needed in order to lower the burden of toxicity, simplify the administration, and above all, to overcome drug resistance selection [6].

Among the HIV-encoded proteins, reverse transcriptase (RT) represents the most exploited target, with more than half of the approved anti-HIV-1 drugs inhibiting this enzyme. RT is a heterodimeric (p66/p51) multifunctional enzyme that acts at the early stage of viral infection by converting the viral single-stranded RNA genome into a double-stranded DNA. This event relies on the concerted activity of the two main distinct associated activities that RT possesses: RNA- and DNA-dependent DNA polymerase (RDDP and DDDP, respectively) activity, that synthesizes the proviral DNA, and ribonuclease H (RNase H) activity, that selectively cleaves the RNA strand within the RNA:DNA heteroduplex replication intermediate along with processing primers [7]. RT heterodimer stability and flexibility are critical requirements for its activity [8].

Considering its pivotal role in virus replication [9,10,11,12], RT-associated RNase H function could represent an alternative promising anti-HIV-1 target. Mutagenesis studies have validated the importance of RNase H activity in the viral replication, since mutations within its domain abolish HIV-1 infectivity [9,10], and in vivo studies confirmed that RNase H inactivation results in non-infectious virus particles [11,12]. During the last three decades, structurally different classes of RNase H inhibitors (RNHIs) have been identified [13,14,15,16], but, in stark contrast to the success achieved with the inhibition of RT-associated DNA polymerase function, no RNase H inhibitor (RNHI) has progressed to the clinic so far. Based on the mechanism of action, RNHIs can be divided in two classes: metal-chelating active site inhibitors and allosteric inhibitors. As already reviewed [13,14,15,16] the majority of RNHIs are characterized by a critical pharmacophore component responsible for the coordination of the two divalent cations present at the active site of the RNase H domain (located at the C-terminus of the p66 subunit) and required for the enzymatic catalysis. A few classes of RNHIs binding outside the RNase H active site have also been identified. Some of them are dual inhibitors active against both the RT-associated enzymatic functions while others emerged as selective RNHIs.

**Figure 1:**
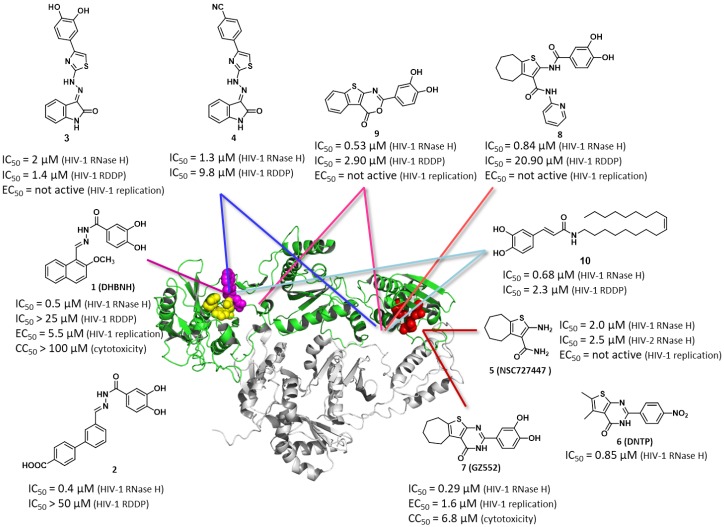
Co-crystal structure of compound **1** (magenta) bound to HIV-1 RT (PDB ID: 2I5J) [17]. Chemical structures of the allosteric RNHIs **1** [17], **2** [18], **3** [19,20], **4** [21], **5** [22,24], **6** [23,24], **7** [25], **8** [27], **9** [28], and **10** [29] reported so far and their predicted allosteric binding sites are also indicated. The IC_50_ value represents the compound concentration that reduces the HIV-1 RT-associated RNase H activity by 50% (HIV-1 RNase H) or the HIV-1 RT-associated RDDP activity by 50% (HIV-1 RDDP); the EC_50_ value represents the compound concentration that inhibits HIV-1 replication by 50%; The CC_50_ value represents the compound concentration that inhibits cell growth by 50%; n.d. indicates not determined value. Yellow spheres, polymerase catalytic site; red spheres, RNase H catalytic site.

Figure 1 summarizes all the chemotypes reported until now as allosteric inhibitors of RNase H, highlighting the binding sites hypothesized by docking studies that also justify the dual action of unselective RNHIs.

*N*-acylhydrazone derivative **1** (DHBNH) was the first allosteric RNHI to be identified. It showed a sub-micromolar selective inhibition that translated well into good anti-HIV activity in cell culture. Co-crystallization of **1** with the HIV-1-RT (PDB 2I5J) highlighted that its binding site is located >50 Å away from the RNase H active site and near both the polymerase active site and the binding pocket recognized by non-nucleoside RT inhibitors [17]. A successive structural optimization study led to the analogue **2**, for whichno binding studies or anti-HIV evaluation has been reported [18].

By using compound **1** as a query for a virtual screening of National Cancer Institute (NCI) libraries, the isatin derivative **3** was identified as a dual inhibitor of RNase H and RDDP activities in the low micromolar range. Docking studies suggested a binding mode similar to that described for compound **1** [19,20]. From the successive exploration of the catechol moiety, the cyano derivative **4** was identified that maintained dual RT inhibition. A combination of biochemical, mutagenesis, and blind docking studies supported the hypothesis that compound **4** exerted its dual inhibition by interacting with two allosteric sites, one placed in the DNA polymerase domain, partially overlapping the NNRTI binding site, and a second one closed to the RNase H active site [21].

A HTS of NCI libraries led to the identification of the thiophene-3-carboxamide derivative **5** (NSC727447) (also known as a vinylogous urea) that emerged as a selective inhibitor of HIV-1 and HIV-2 RNase H with an IC_50_ value of 2.0–2.5 µM [22]. An anti-HIV-1 activity at the same concentration was also reported for this compound, albeit combined with a low selectivity index (SI = 4). Successive SAR studies on commercially available thiophene-3-carboxamide analogues led to the identification of the sub-micromolar RNHI thienopyrimidinone **6** (DNTP) [23]. Mutagenesis and molecular modeling studies suggested that compounds **5** and **6** recognize an allosteric site located at the interface between p66 RNase H domain and p51 thumb subdomain, thus hampering the subunit flexibility essential for the RNA:DNA hybrid binding [24]. The synthesis of further thienopyrimidinones led to the catechol derivative **7** (GZ552), for which the inhibition of RNase H translated into HIV-1 replication inhibition, even if at concentrations near cytotoxicity [25]. Differential scanning fluorimetry demonstrated its ability to destabilize the RT heterodimer, thus confirming an allosteric binding at the interface between p51 and p66 RT subunits.

Based on the structural similarity with compound **5**, a series of cycloheptathiophene-3-carboxamide derivatives, initially identified by us as anti-influenza agents able to disrupt the PA-PB1 subunits interaction of the influenza RNA polymerase [26], has been repurposed for HIV-1 RNase H activity. From the initial screening, compounds endowed with good inhibitory activity emerged, prompting the structural optimization that led to identify the catechol derivative **8**. With an IC_50_ value of 0.84 µM, compound **8** ranked among the most active allosteric RNHIs [27]. Docking simulations suggested that its binding site is located under the RNase H catalytic site, with the p66 residue Q500, known to interact with the RNA strand within the RNA:DNA duplex, as a key residue for binding [27]. A successive optimization campaign created a series of tricyclic and bicyclic RNHIs, among which the benzothienooxazinone **9** exhibited the most potent inhibitory activity against both RNase H and RDDP functions, with IC_50_ values of 0.53 and 2.90 µM, respectively [28]. Mutagenesis and docking analyses explained its dual behaviour thanks to the ability to recognize two different pockets, the first located closed to the DNA polymerase catalytic centre (partially overlapping the NNRTIs binding site) and the second closed to the RNase H active site and near the p66-p51 interface [28].

Starting from the antiviral activity of an *Ocimum sanctum* leaves extract and then by synthesizing some analogues, the *N*-oleylamide derivative of caffeic acid 10 was identified with dual RT inhibition in the low micromolar range. Docking experiments located compound 10 in the same binding site identified for compound 1, along with a second binding site placed below the RNase H catalytic site between p66 and p51 subunits [29].

In this work, the repurposing of in-house anti-influenza derivatives based on the 1,2,4-triazolo[1,5-*a*]pyrimidine (TZP) scaffold followed by a successive structural optimization study permitted us to identify allosteric inhibitors of the RNase H function active in the low micromolar range.

## 2. Results and Discussion

### 2.1. Structural Exploration of the 1,2,4-Triazolo[1,5-a]pyrimidine Scaffold

The present work started by testing two compounds belonging to a class of in-house anti-influenza derivatives characterized by the 4,7-dihydro-TZP scaffold [30]. Both the compounds **11a** and **11b** (Table 1) inhibited the RNase H with IC_50_ values in the micromolar range (IC_50_ = 17.7 and 13.1 µM, respectively), thus representing valid hit compounds. Since we have previously found that TZP derivatives are synthetically more manageable than the 4,7-dihydro-TZPs [30], a first structural modification entailed the aromatization of the **11b** core, synthesizing compound **12b**. Preserving the same anti-RNase H activity, the aromatic TZP core was maintained in all successive analogues (Table 1). The C-2 phenyl ring was explored by introducing substituents with different electronic and steric properties, including the catechol moiety previously emerged as a particularly suitable in imparting RNase H inhibitory activity [27,28] (compounds **12c**–**g**). Then, by maintaining the same substituents at C-2 position, the TZP scaffold was modified by interchanging the methyl group and the phenyl ring at C-5 and C-7 positions, as in compounds **13c**–**g**, as well as by maintaining a methyl group at both C-5 and C-7 positions, as in compounds **14b**–**g**. Among the whole set of derivatives, only compounds bearing the catechol moiety exhibited good RNase H inhibitory activity. Thus, further modifications were undertaken by fixing the catechol group on the TZP core. In particular, a phenyl ring at both C-5 and C-7 positions characterized derivative **15g**, while the catechol moiety was shifted to C-7 position of the scaffold in compound **17h**. The di-amide compound **16hh**, obtained as a side product during the synthesis of compound **17h**, was also tested for the biological activity. Finally, the presence of an inverse amide linkage between the TZP core and the C-2 catechol ring was investigated in the couple of positional isomers **18g** and **19g**.

### 2.2. Chemistry

**Scheme 1:**
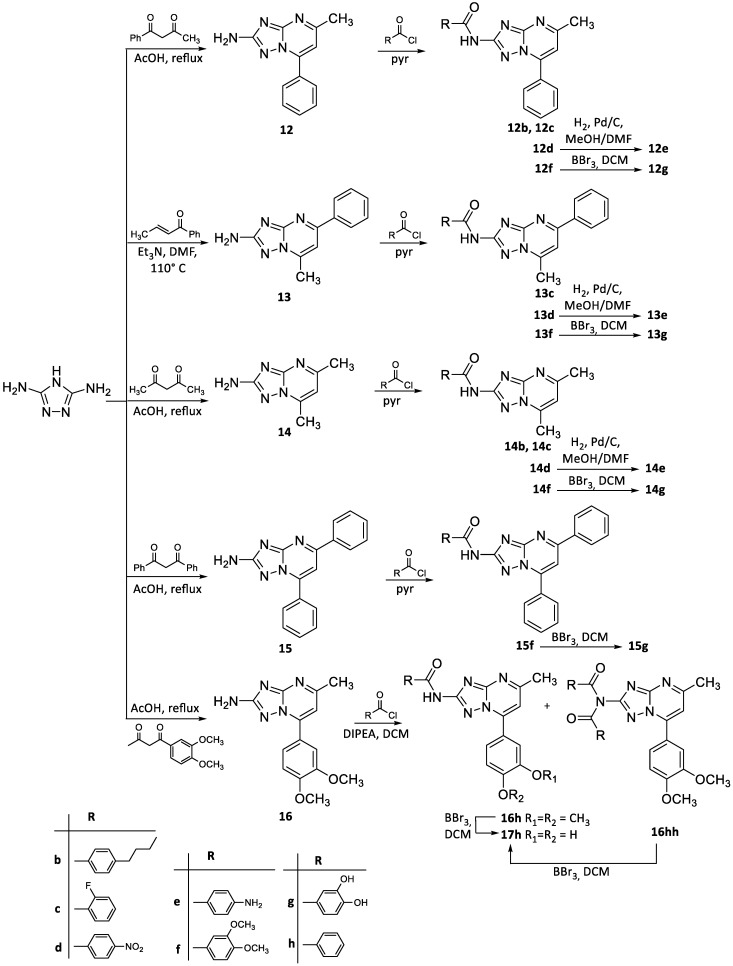
Synthetic routes to compounds **12b–g**, **13c-g**, **14b–g**, **15g**, **17h**, and **16hh**.

The synthesis of the 2-amino-1,2,4-triazolo[1,5-*a*]pyrimidine derivatives **12b**–**g**, **13c**–**g**, **14b**–**g**, **15g**, **16hh** and **17h** was accomplished starting with the preparation of the 2-amino synthons **12**–**16**, as shown in Scheme 1. Synthons **12** [31] and **13** [31] were synthesized via reaction of 3,5-diaminotriazole with 1-phenylbutane-1,3-dione and 1-phenylbut-2-en-1-one, respectively [31]. Synthons **14** [32] and **15** [33] were prepared through a more efficient procedure than that already published, entailing the reaction of 3,5-diamino-1,2,4-triazole with the appropriate β-diketone in glacial acetic acid at reflux. Accordingly, by following the same procedure synthon **16** was also obtained. The successive amidation reaction of synthons **12**–**15** with the appropriate benzoyl chloride in dry pyridine afforded the target compounds **12b**–**d**, **12f**, **13c**–**d**, **13f**, **14b**-**d** and **14f**, respectively. Conversely, in the case of synthon **16**, probably due to its low solubility in pyridine, the amidation reaction with benzoyl chloride was accomplished in dry freshly distilled DCM, in the presence of DIPEA, leading not only to compound **16h** but also to the diamide derivative **16hh**. The *p*-aminophenyl derivatives **12e**, **13e**, and **14e** were then obtained by catalytic hydrogenation in presence of palladium on carbon of nitro precursors **12d**, **13d**, and **14d**, respectively, while *O*-demethylation of dimethoxy derivatives **12f**, **13f**, **14f**, **15f**, and **16h** was performed with BBr_3_ in DCM providing the corresponding dihydroxy derivatives **12g**, **13g**, **14g**, **15g**, and **17h**, respectively. The same *O*-demethylation reaction conducted with compound **16hh** did not furnish its corresponding 3,4-dihydroxyphenyl derivative, but led again to the monoamide derivative **17h**.

**Scheme 2:**
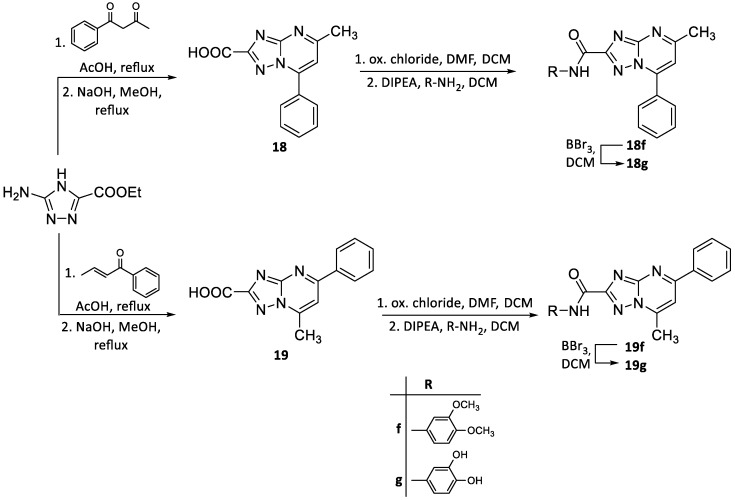
Synthetic routes to compounds **18g** and **19g**.

As outlined in Scheme 2, the synthesis of 2-carboxyamide-1,2,4-triazolo[1,5-*a*]pyrimidine derivatives **18g** and **19g** was accomplished starting from the carboxylic acid synthons **18** [31] and **19 [31]**, synthesized as already described in the literature [31] by reacting ethyl 5-amino-1,2,4-triazole-3-carboxylate [34] with 1-phenylbutane-1,3-dione and 1-phenylbut-2-en-1-one, respectively, and then hydrolysing the ethyl esters groups under basic conditions. Thus, coupling reaction between their corresponding carbonyl chlorides with 3,4-dimethoxyaniline performed in dry DCM in the presence of DIPEA, led to intermediates **18f** and **19f**, which after the O-demethylation furnished the target compounds **18g** and **19g**, respectively.

### 2.3. Evaluation of RNase H and RDDP Inhibitory Activity

A total of 24 variously functionalized TZP derivatives were evaluated for their ability to inhibit the HIV-1 RT-associated RNase H (Table 1). In each experiment, the active site RNHI **RDS1643** [35] and the allosteric RNHI **5** (NSC727447), specially re-synthesized by us for this purpose, were tested for comparative purposes.

From the anti-RNase H activity evaluation clearly emerged how among the various structural modifications made around the TZP scaffold only rare peculiar substitutions were suitable to impart inhibitory activity. While the aromatization of the 4,7-dihydro-TZP derivative **11b** (IC_50_ = 13.1 µM), permitted to maintain the same anti-RNase H activity in compound **12b** (IC_50_ = 12.3 µM), the replacement of the phenyl ring at the C-7 position with a second methyl group, as in compound **14b**, induced a complete loss of activity, suggesting that a C-7 phenyl ring at the TZP scaffold may have a essential role in the interaction with the target. Considering the substitution exploration of the C-2 phenyl ring in the set of compounds **12b**–**g**, only the catechol moiety was able to impart RNase H inhibitory activity, as in compounds **12g** that, with an IC_50_ of 0.8 µM, emerged as more potent than the starting compounds **11a** and **11b**. Analogously, when the phenyl ring and the methyl group were interchanged at C-5 and C-7 positions (compunds **13c**–**g**), or a methyl group as well as a phenyl ring were maintained at both the C-5 and C-7 positions (compounds **14b**–**g** and **15g**, respectively), only the C-2 catechol-decorated TZP derivatives **13g**, **14g**, and **15g** emerged endowed with RNase H inhibitory activity (IC_50_ = 3.5, 6.23 and 1.86, respectively). This suggested that, independently by the substitution pattern around the TZP scaffold, the cathecol moiety is the sole able to impart potent RNase H inhibitory activity. Additional SAR insights are that the C-5 methyl group and C-7 phenyl ring emerged as the most suitable combination for the RNase H inhibition (compound **12g**, IC_50_ = 0.8 µM), better than the interchanged C-5 phenyl ring and C-7 methyl group pattern (compound **13g**, IC_50_ = 3.5 µM); the complete removal of the phenyl ring on the TZP core decreases even more the inhibitory activity (compound **14g**, IC_50_ = 6.23 µM); while the presence of a second phenyl ring does not interfere with the RNase H inhibitory activity (compound **15g**, IC_50_ = 1.86 µM). With an IC_50_ of 0.41 µM, compound **18g** emerged as the most potent RNHI of the series, confirming the suitability of the C-7 phenyl ring even when the amide linkage was inverted. As further confirmation, its C-5 phenyl regioisomer **19g** showed a markedly decreased anti-RNase H activity. Finally, the critical role of catechol as peculiar substituent to achieve potent anti-RNase H activity was also confirmed by compound **17h** (IC_50_ = 1.13 µM), in which the catechol was moved to C-7 position of the scaffold. Unexpectedly, compound **16hh**, characterized by a bulky di-amide moiety at C-2 position coupled with a 3,4-dimethoxyphenyl at C-7 position, maintained the ability to inhibit the RNase H activity in the low micromolar range. This compound, beside to confirm the appropriateness of a substituted phenyl as C-7 substituent, highlighted how a bulkier substituent is still tolerated at the C-2 position.

All the compounds were also tested for their activity against the RDDP, using the non nucleoside RDDP inhibitor **efavirenz** as a control. With only one exception, all the tested TZPs showed the inability to inhibit the DNA polymerase function at the highest tested concentration of 100 µM. Only the diphenyl derivative **15g** exhibited RDDP inhibitory activity with IC_50_ value of 20.5 ± 4.1 µM, hence with a 10-fold weaker potency than that demonstrated against the RNase H.

### 2.4. Mg^2+^ Coordination Analysis

As it has been widely reported, all the RNHIs interacting with the active site are endowed with the ability to coordinate the divalent cations required for the enzymatic catalysis. Thus, to investigate a possible binding to the RNase H catalytic domain, the most active compounds **12g**, **13g**, **14g**, **15g**, **16hh**, **17h**, and **18g** were selected for Mg^2+^ ions coordination analysis. According to the UV spectra recorded in the absence and in presence of MgCl_2_, differently from active site RNHIs such as **RDS1643**, none of the TZP derivatives displayed chelation ability, since no shift was observed at the maximum of absorbance (hypsochromic effect) (see Supplementary Materials, Figure S1). These results clearly suggested that the TZP scaffold represents a new chemotype of RNHIs binding through an allosteric RNase H site.

### 2.5. In Silico Studies

**Figure 2:**
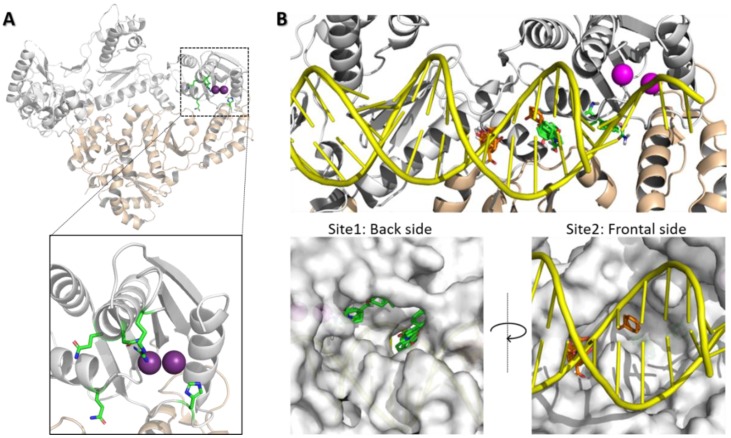
(**A**) Catalytic center of the RNase H domain. Residues involved in the protein-duplex stabilization and surrounding the catalytic center are highlighted in stick. (**B**) Position of the FTMap identified pockets. Green sticks, Site1 probes; orange sticks, Site2 probes; magenta spheres, Mg^2+^ ions; white cartoon, p66; wheat cartoon, p55; yellow cartoon, DNA:DNA duplex. The figure was obtained using the 3LP1 structure. The DNA duplex coordinates were retrivied from 5J2M crystal structure.

The Mg^2+^ ions coordination analysis suggested that TZP derivatives could act as allosteric inhibitors. It is well known that allosteric binding usually involves pockets that are distant 10–20 Å from key regions for protein activity [36]. Thus, we focused our attention on possible binding sites located around the catalytic center of the RNase H domain. Of note, in the explored region, various residues involved in duplex-protein binding [7] were present (Figure 2A). Allosteric inhibitors acting in this region can prevent the catalytic activity and hamper a stable duplex-protein interaction.

In order to achieve insights on the TZPs binding to the RNase H domain, in silico studies were performed with the aim to analyse the RNase H domain and identify plausible ligand-binding pockets and thus suggesting the key intermolecular interactions.

In RCSB Protein Data Bank (PDB) [37] 326 crystal structures reporting the 3D coordinates of HIV-RT are freely available. Among them, we decided to select only 18 crystal structures, corresponding to the HIV-1 RT with no engineered mutations (see Selection of crystal structure subset in the Materials and Method section for details) and having a resolution ≤2.5 Å to submit them to FTMap analysis [38].This software identifies ligand-binding hotspots on the protein surface by predicting the possible distribution of small organic probes on the protein surface. The FTMap results were evaluated on the base of i) the total number of probes accommodated in the same pocket (#Pprobes), ii) the presence in the pocket of at least one aromatic probe, given the presence of at least one aromatic ring as substituent in the TZP derivatives and iii) the proximity of predicted pocket to the RNase H catalytic site. FTMap analysis highlighted the presence of two possible pockets in the explored protein region (named Site1 and Site2 in Figure 2 and Table S1, see Supplementary Materials); however, Site1 clearly emerged as the most interesting site. Indeed, Site1 was present in all the analysed conformations and it generally corresponded to a higher #Pprobe (mean of #Pprobe of 12.9 and 7.5 for Site1 and Site2, respectively; Table S1). Of note, these results are in line with two previous studies performed on HIV-1 RT suggesting the same Site1 as potential ligand-binding site [39,40]. As highlighted in Figure 2B (Site1: Back side), Site1 is a pocket not exploited by any previous RNHIs, located below the RNase H active site and accessible only from the back side of the protein, opposite to that one interested by the duplex binding. Due to the promising FTMap results, the following analysis were focused on this pocket (i.e., Site1).

**Figure 3:**
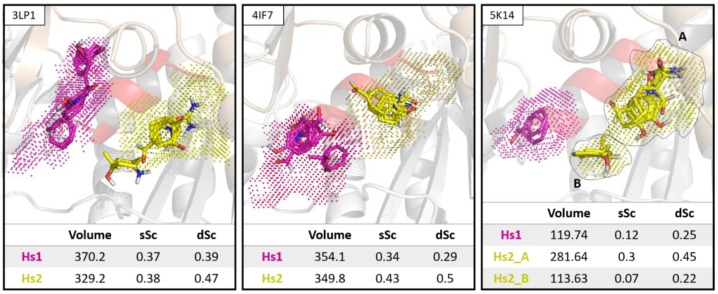
Overlap between FTMap probes (stick representation) and subpockets identified by DogSiteScorer (dots representation) using 3LP1, 4IF7 and 5K14 Site1 conformations. sSc: Site Score; dSc: Drug Score. Volume is expressed in Å.

Three Site1 conformations were selected as significantly populated regions (i.e., regions with a #Pprobes ≥ 20 and at least one aromatic probe) in the 3LP1, 4I7F and 5K14 crystal structures. In all the three Site1 conformations, two different hot spot regions were present and will be hereafter called hot spot 1 (Hs1) and hot spot 2 (Hs2). The druggability of the identified Hs regions was assessed using DogSiteScorer [41], that evaluates a pocket in terms of Simple Score (sSc) and Drug Score (dSc), where the higher the value, the better the druggability. Interestingly, while Hs2 (yellow in Figure 3) shared a conserved shape among the three structures, a deeper exploration of Hs1 (magenta in Figure 3) in 3LP1 conformation was obtained by the FTMap probes. Of note, the predicted Hs regions were in close contact with the α-helix 14 in which is located the residue Q500, essential for duplex binding. Considering both Hs regions of Site1, a low druggability was obtained for 5K14 conformation (i.e., low values of volume, sSc and dSc). By contrast, a promising druggability was obtained for 3LP1 and 4I7F, the former characterized by the most druggable Hs1, the latter by the most druggable Hs2 (Figure 3). Given these results, both 3LP1 and 4I7F Site1 conformations were used for further in silico experiments.

Indeed, to get insight about the potential interactions established by the TZP derivatives into Site1, the most potent compounds (i.e., **12g**, **13g**, **15g**, **17h** and **18g**) were submitted to docking simulations. In addition, given the high structural similarity but different activity between derivatives **13g** and **19g** (IC_50_ of 3.5 and 43.1 µM, respectively), the latter was also included in this study.

**Figure 4:**
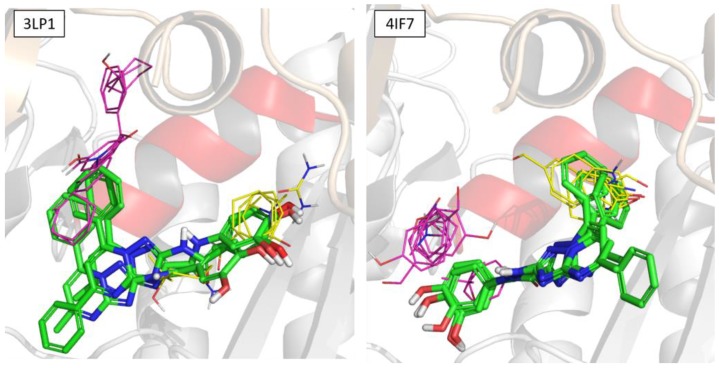
Superimposition of docking poses for derivatives **12g**, **15g**, and **18g** (green) for 3LP1 and 4IF7 proteins. Magenta lines, Hs1 probes; yellow lines, Hs2 probes; red cartoon, α-helix 14.

For each protein, a conserved binding mode was obtained for compounds **12g**, **15g**, and **18g** (Figure 4). In 3LP1 Site1 conformation, the C-2 catechol moiety of TZP derivatives was always placed into the Hs2, while the C-7 ring was oriented into Hs1. This overall orientation was respected also for compound **17h** (see Supporting Materials, Figure S2) although the catechol moiety is placed at C-7 position.

**Figure 5:**
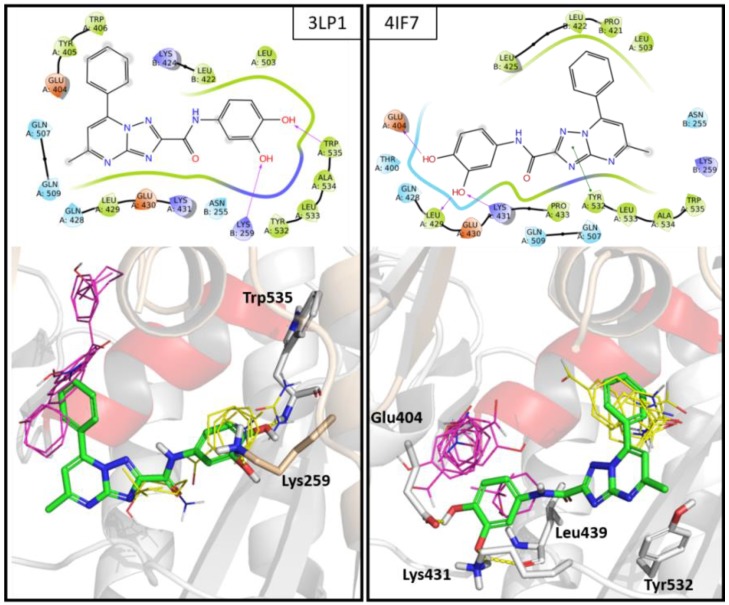
2D and 3D representation of **18g** binding mode produced by docking studies on 3LP1 and 4I7F structure. Magenta lines, Hs1 probes; yellow lines, Hs2 probes; red cartoon, α-helix 14.

The predicted binding mode of derivative **18g** as representative is reported in Figure 5. In this compound, the TZP core was able to orient the two aromatic rings into Hs1 and Hs2. In particular, the catechol moiety established two hydrogen bonds with Trp535 and Lys259 in Hs2 and the phenyl ring was placed into Hs1. Additionally, **18g** established hydrophobic interactions with Tyr405, Gln428, Leu503, Gln507, Gln509 and Trp535. Interestingly, the predicted activity (Ki_pred_) was in line with the experimental IC_50_ value (Ki_pred_ = 0.37 µM; IC_50_ = 0.4 µM; Table S2). By contrast, the 4I7F conformation provided binding poses for the analysed derivatives in which the C-2 catechol moiety was placed in Hs1 and the C-7 phenyl ring was oriented into Hs2. Even though the catechol moiety was involved in a complex H-bond network, Hs1 region defined by the FTmaps probes was only marginally occupied by the compound (Figure 5). Of note, no reliable pose was obtained for the active TZP derivative **17h**.

**Figure 6:**
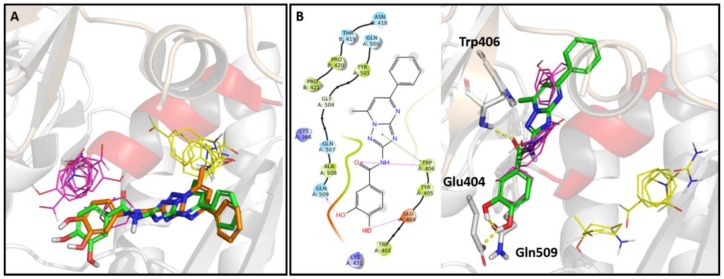
(**A**) Superimposition of docking poses for derivatives **13g** (green) and **19g** (orange) in 4IF7 protein. (**B**) 2D and 3D representation of **13g** (green) binding mode produced by docking studies on 3LP1 structure. Magenta lines, Hs1 probes; yellow lines, Hs2 probes; red cartoon, α-helix 14.

However, the most interesting results were obtained for compounds **13g** (IC_50_ = 3.5 µM) and **19g** (IC_50_ = 43.1 µM). For 4I7F protein, molecular docking returned a reliable and high-conserved binding pose for both compounds (Figure 6 and Table S2) similar to that suggested for compounds **12g**, **15g** and **18g**. These results did not explain the different inhibitory activity of the congener compounds (i.e., **13g** and **19g**). By contrast, the same methodology on 3LP1 structure reproduced the binding mode of the sole compound **13g**, in a completely different orientation with respect to the binding mode of **18g** (Figure 6). Of note, **13g** was completely placed in Hs1 and interacted with regions that were accessible only in the 3LP1 conformation (Figure 3). In particular, the catechol moiety established two hydrogen bonds with Lys431 and Gln509, while the carboxy amide group made a hydrogen bond with Trp406. No poses were retained for compound **19g**, highlighting as, in line with the RNase H inhibitory activity, the inversion of the amide linkage in compound **13g** penalised the docking results in 3LP1 protein.

The overall docking results obtained for 3LP1 structure suggested that the occupation of the Hs1 could be a key feature to have an efficient ligand binding on Site1. In the analysed compounds **12g**, **15g**, **17h**, and **18g** the Hs1 was well explored by using the C-7 moiety. In **13g** the absence of C-7 phenyl ring was mitigated by a different compound orientation that led to Hs1 filling. In this alternative orientation, the amide inversion was detrimental for ligand binding. Indeed, no poses were retained for compound **19g**.

The results of our docking studies provided useful information about the key interactions of the analysed TZP derivatives with each one of the two selected conformations (i.e., 3LP1 and 4I7F) of Site1. However, the in silico studies showed that 3LP1 conformation can give a more realistic rationalization of TZP biological activity, suggesting a link between Hs1 occupation and allosteric inhibition activity. The differences in the identified hot spot regions for Site1 conformations (Figure 3) can mirror a pocket flexibility that, coupled with the near proximity of this site to α-helix **14** and catalytic site, is in line with the intrinsic flexibility of a putative allosteric pocket.

### 2.6. Site Directed Mutagenesis

To experimentally verify the binding mode suggested by computational studies, alanine substitution was introduced at residue Trp535 within the HIV-1 RNase H domain. Such substitution, would abrogate the interaction predicted between Trp535 and compound **18g** in the docking model (3LP1 crystal). Compound **18g** was tested against the RNase H activity of Trp535Ala mutant, showing an IC_50_ >100 μM. The drastic reductions of inhibitory potency, >244 fold of difference, proved that the Trp535 is essential for **18g** inhibition validating the binding mode.

### 2.7. Anti-HIV-1 Activity

Compounds **11b**, **12g**, **13g**, **15g**, **17h**, and **18g** were tested for their anti-HIV-1 (III_B_ strain) and HIV-2 (ROD strain) activity in acutely infected MT-4 cells, assaying in parallel their cytotoxicity in the same cell line. Unfortunately, none of the compounds having RNase H inhibition showed anti-HIV activity at concentrations lower than those cytotoxic (CC_50_ values ranging from 4.58 to 29.28 µM, with the exception of compound **12g** that was devoid of any cytotoxic effect showing CC_50_ = 112.36 µM).

Additional studies were performed in order to justify the lack of antiviral activity of these new RNHIs. Firstly, we wondered if the tested compounds would be able to efficiently cross cell membranes. Thus, Caco-2 cell a permeability prediction, using the Qikprop tool, [42] was generated for the most active inhibitor **18g**, suggesting its low capability to permeate through the membrane (QPPCaco = 116.7 nmol/s, with QPPCaco values <25 nmol/s corresponding to low permeability and values > 500 nmol/s being associated to great permeability), thus providing a possible explanation for the lack of antiviral activity for this compound

Then, we decided to perform dedicated studied in order to discard the possibility that the RNase H inhibitory activity detected in the in vitro assays could be an artefact. Indeed, the presence of the catechol moiety emerged particularly stringent to achieve RNase H inhibitory activity in the TZP derivatives analogously to many of the compounds previously reported. However, catechol has been associated to the well-known Pan-Assay Interference Compounds (PAINS) family [43,44,45]. PAINS compounds may explicate assay interference or promiscuous behaviour through metal chelation, compound fluorescence, redox activity, cysteine oxidation, or chemical aggregation [43,45].

Thus, the intrinsic fluorescence of the catechol derivatives **12g**, **13g**, **14g**, **15g**, **17h**, and **18g** has been determined to exclude an interference in the readout of the RNase H inhibition assay. As shown in Figure S3 (Supplementary Materials), all the compounds showed no significant fluorescence at the excitation/emission wavelength (490/528 nm) used for the product quantification of the enzymatic assay. Moreover, since all the catechol derivatives emerged unable to chelate Mg^2+^ ions, as reported above in Figure S1 (Supporting Materials), even the metal chelation mechanism can be excluded as possible PAINS activity. Finally, by using the ZINC15 remover filter [46] none of the catechol-decorated TZP derivatives was found as potential aggregator, thus discarding also the hypothesis of chemical aggregation.

## 3. Materials and Methods

### 3.1. Chemistry

#### 3.1.1. General Synthesis Methods and Analysis

All starting materials were commercially available unless otherwise indicated. Commercially available starting materials, reagents, and solvents were used as supplied. All reactions were routinely checked by TLC on silica gel 60F254 (Merck, Darmstadt, Germany) and visualized by using UV or iodine. Flash column chromatography was performed on Merck silica gel 60 (mesh 230–400). After extraction, organic solutions were dried over anhydrous Na_2_SO_4_, filtered, and concentrated with a Büchi rotary evaporator under reduced pressure. Yields are of purified isolated products and were not optimized. Melting points were determined in capillary tubes (Electrothermal Mod. 9100, Büchi, Milan, Italy) and are uncorrected. HRMS spectra were registered on a 6540 UHD Accurate Mass Q-TOF LC/MS-HPLC 1290 Infinity system (Agilent Technologies Santa Clara, CA, USA). Purity of the target compounds was determined by LC/MS on an Agilent Technologies 6550 iFUNNEL Q-TOF equipped with a HPLC 1290 Infinity with DAD detector and evaluated to be higher than 95%. HPLC conditions to assess the purity of final compounds were as follows: column, AERIS Widepore C4 (Phenomenex, Bologna, Italy) 4.6 mm × 100 mm (6.6 μm); flow rate, 0.85 mL/min; acquisition time, 10 min; DAD 190−650 nm; oven temperature, 30 °C; gradient of acetonitrile in water containing 0.1% of formic acid (0−100% in 10 min). ^1^H-NMR and ^13^C-NMR spectra were recorded on Avance DPX-200 and Avance DRX-400 MHz instruments (Bruker Milano, Italy) using residual solvent peaks such as that of dimethylsulfoxide (δ = 2.48) as an internal standard. Chemical shifts were recorded in ppm (δ) and the spectral data are consistent with the assigned structures. The spin multiplicities are indicated by the symbols s (singolet), d (doublet), t (triplet), q (quartet), m (multiplet), and bs (broad singolet).

##### General Procedure for C-2 Amidation (Method A)

To the solution of the appropriate synthons (1.0 equiv) in dry pyridine the appropriate benzoyl chloride (2.0 equiv) was added dropwise. The reaction mixture was maintained at room temperature until no starting material was detected by TLC (1–24 h). After cooling, the reaction mixture was poured into ice/water, yielding a precipitate which was filtered and purified as described below.

*4-Butyl-N-(5-methyl-7-phenyl-[1,2,4]triazolo[1,5-a]pyrimidin-2-yl)benzamide* (**12b**). The title compound was prepared starting from **12** [31] through Method A (24 h) by using 4-butylbenzoyl chloride, and purified by flash chromatography eluting with CHCl_3_/MeOH (99:1), in 25% yield as white solid; mp 157-158 °C. ^1^H-NMR (DMSO-*d*_6_, 400 MHz): δ 0.85 (t, *J* = 7.3 Hz, 3H, CH_2_*CH_3_*), 1.20-1.35 (m, 2H, *CH_2_*CH_3_), 1.50-1.60 (m, 2H, *CH_2_*CH_2_CH_3_), 2.55-2.70 (m, 5H, C*CH_2_* and CH_3_), 7.30 (d, *J* = 8.1 Hz, 2H, aromatic CH), 7.45 (s, 1H, H-6), 7.55-7.65 (m, 3H, aromatic CH), 7.90 (d, *J* = 8.1 Hz, 2H, aromatic CH), 8.15-8.25 (m, 2H, aromatic CH), 11.20 (s, 1H, NHCO); ^13^C-NMR (DMSO-*d*_6_, 101 MHz): δ 14.1, 22.1, 24.9, 33.1, 35.0, 110.1, 128.5, 128.6, 129.0, 129.8, 130.1, 131.3, 131.8, 145.8, 147.3, 155.1, 160.3, 164.8, 165.1.

*2-Fluoro-N-(5-methyl-7-phenyl[1,2,4]triazolo[1,5-a]pyrimidin-2-yl)benzamide* (**12c**). The title compound was prepared starting from **12** [31] through Method A (2h) by using 2-fluorobenzoyl chloride, and purified by crystallization by cyclohexane/EtOAc, in 9% yield as white solid; mp 292-302 °C. ^1^H-NMR (DMSO-*d*_6_, 400 MHz): δ 2.75 (s, 3H, CH_3_), 7.25-7.35 (m, 2H, aromatic CH), 7.45 (s, 1H, H-6), 7.50-7.60 (m, 5H, aromatic CH), 8.10-8.15 (m, 2H, aromatic CH), 11.45 (s, 1H, NHCO).

*N-(5-Methyl-7-phenyl-[1,2,4]triazolo[1,5-a]pyrimidin-2-yl)-4-nitrobenzamide* (**12d**). The title compound was prepared starting from **12** [31] through Method A (1h) by using 4-nitrobenzoyl chloride, and purified by flash chromatography eluting with DCM/MeOH (98:2), in 29% yield as light yellow solid; mp 290–293 °C. ^1^H-NMR (DMSO-*d*_6_, 200 MHz): δ 2.75 (s, 3H, CH_3_), 7.45 (s, 1H, H-6), 7.55–7.65 (m, 3H, aromatic CH), 8.10-8.20 (m, 4H, aromatic CH), 8.30 (d, *J* = 8.8 Hz, 2H, aromatic CH), 11.55 (s, 1H, NHCO).

*4-Amino-N-(5-methyl-7-phenyl-[1,2,4]triazolo[1,5-a]pyrimidin-2-yl)benzamide* (**12e**). To a solution of **12d** (0.12 g, 0.32 mmol) in a MeOH/DMF (1:2) mixture (12 mL) Pd/C 10% (0.24 g) and ammonium formate (0.10 g, 1.60 mmol) were added. The reaction mixture was maintained at rt for 4 h and then filtered on celite. The filtrate was evaporated to dryness and the crude product was purified by flash chromatography eluting with CHCl_3_/MeOH (98:2), to give **12e** (0.044 g, 40%) as light yellow solid; mp 179-202 °C. ^1^H-NMR (DMSO-*d*_6_, 400 MHz): δ 2.70 (s, 3H, CH_3_), 5.75 (s, 2H, NH_2_), 6.50 (d, *J* = 8.4 Hz, 2H, aromatic CH), 7.45 (s, 1H, H-6), 7.55-7.65 (m, 3H, aromatic CH), 7.75 (d, *J* = 8.4 Hz, 2H, aromatic CH), 8.15-8.25 (m, 2H, aromatic CH), 11.00 (s, 1H, NHCO).

*3,4-Dimethoxy-N-(5-methyl-7-phenyl-[1,2,4]triazolo[1,5-a]pyrimidin-2-yl)benzamide* (**12f**). The title compound was prepared starting from **12** [31] through Method A (2h) by using 3,4-dimethoxybenzoyl chloride, and purified by flash chromatography eluting with DCM/MeOH (95:5), in 53% yield as light yellow solid; mp 131–151 °C. ^1^H-NMR (DMSO-*d_6_*, 400 MHz): δ 2.65 (s, 3H, CH_3_), 3.80-3.85 (s, 6H, OCH_3_), 7.05 (d, J = 8.4 Hz, 1H, aromatic CH), 7.45 (s, 1H, H-6), 7.55–7.70 (m, 5H, aromatic CH), 8.10-8.20 (m, 2H, aromatic CH), 11.25 (s, 1H, NHCO).

##### General Procedure for *O*-demethylation Reaction (Method B)

To a solution of the appropriate methoxy derivative (1.0 equiv) in dry CH_2_Cl_2_, 1M solution of BBr_3_ in DCM (6.0 equiv) was added dropwise maintaining the temperature at 0 °C. The reaction mixture was stirred at room temperature until no starting material was detected by TLC (3–16h), and then quenched with MeOH and water. The organic solvent was removed under vacuum affording a residue, which was filtered and purified as described below.

*3,4-Dihydroxy-N-(5-methyl-7-phenyl-[1,2,4]triazolo[1,5-a]pyrimidin-2-yl)benzamide* (**12g**). The title compound was prepared starting from **12f** through Method B (3h) and purified by flash chromatography eluting with CHCl_3_/MeOH (97:3), in 22% yield as light yellow solid; mp 285–288 °C. ^1^H-NMR (DMSO-*d*_6_, 400 MHz): δ 2.65 (s, 3H, CH_3_), 6.80 (d, *J* = 8.8 Hz, 1H, aromatic CH), 7.35-7.40 (m, 2H, aromatic CH), 7.45 (s, 1H, H-6), 7.55–7.65 (m, 3H, aromatic CH), 8.15–8.25 (m, 2H, aromatic CH), 9.25 and 9.75 (s, each 1H, OH), 11.00 (s, 1H, NHCO). HRMS (ESI) *m/z* [M + Na]+ calcd for C_19_H_15_N_5_O_3_ 384.10671, found 384.10689.

*2-Fluoro-N-(7-methyl-5-phenyl-[1,2,4]triazolo[1,5-a]pyrimidin-2-yl)benzamide* (**13c**). The title compound was prepared starting from **13** [31] through Method A (16h) by using 2-fluorobenzoyl chloride, and purified by crystallization by EtOH, in 36% yield as light yellow crystals; mp 165–169 °C. ^1^H-NMR (DMSO-*d*_6_, 400 MHz): δ 2.75 (s, 3H, CH_3_), 7.25-7.35 (m, 2H, aromatic CH), 7.50–7.60 (m, 4H, aromatic CH), 7.65 (t, *J* = 7.3 Hz, 1H, aromatic CH), 7.85 (s, 1H, H-6), 8.10– 8.20 (m, 2H, aromatic CH), 11.50 (s, 1H, NHCO); ^13^C-NMR (DMSO-*d*_6_, 101 MHz): δ 17.4, 107.3, 116.5(*J_C-F_* = 86 Hz), 124.4 (*J_C-F_* = 59 Hz), 124.9, 127.7, 129.4, 130.4, 131.5, 133.3 (*J_C-F_* = 33 Hz), 136.5, 148.2, 154.9, 158.2, 1597, 160.2, 160.7, 162.7

*N-(7-Methyl-5-phenyl-[1,2,4]triazolo[1,5-a]pyrimidin-2-yl)-4-nitrobenzamide* (**13d**). The title compound was prepared starting from **13** [31] through Method A (1 h) by using 4-nitrobenzoyl chloride, and purified by crystallization by EtOH, in 96% yield as white crystals; mp 316-318 °C. ^1^H-NMR (DMSO-*d*_6_, 400 MHz): δ 2.75 (s, 3H, CH_3_), 7.50–7.60 (m, 3H, aromatic CH), 7.90 (s, 1H, H-6), 8.15–8.25 (m, 4H, aromatic CH), 8.35 (d, *J* = 8.5 Hz, 2H, aromatic CH), 11.75 (s, 1H, NHCO).

*4-Amino-N-(7-methyl-5-phenyl-[1,2,4]triazolo[1,5-a]pyrimidin-2-yl)benzamide* (**13e**). The title compound was prepared starting from **13d** by using the same procedure used for the synthesis of **12e** (1h), and purified by crystallization by EtOH/DMF mixture, in 8% yield as light yellow crystals; mp 318-319 °C. ^1^H-NMR (DMSO-*d*_6_, 400 MHz): δ 2.75 (s, 3H, CH_3_), 5.75 (s, 2H, NH_2_), 6.50 (d, *J* = 8.6 Hz, 2H, aromatic CH), 7.45-7.55 (m, 3H, aromatic CH), 7.70 (d, *J* = 8.4 Hz, 2H, aromatic CH), 7.85 (s, 1H, H-6), 8.15-8.25 (m, 2H, aromatic CH), 11.00 (s, 1H, NHCO).

*3,4-Dimethoxy-N-(7-methyl-5-phenyl-[1,2,4]triazolo[1,5-a]pyrimidin-2-yl)benzamide* (**13f**). The title compound was prepared starting from **13** [31] through Method A (4 h) by using 3,4-dimethoxybenzoyl chloride, and purified by treatment with Et_2_O, in 50% yield as light yellow solid; mp 175–179 °C. ^1^H-NMR (DMSO-*d*_6_, 400 MHz): δ 2.75 (s, 3H, CH_3_), 3.75 and 3.80 (m, each 3H, OCH_3_), 7.10 (d, *J* = 8.4 Hz, 1H, aromatic CH), 7.50-7.60 (m, 3H, aromatic CH), 7.60–7.70 (m, 2H, aromatic CH), 7.90 (s, 1H, H-6), 8.15–8.25 (m, 2H, aromatic CH), 11.25 (s, 1H, NHCO).

*3,4-Dihydroxy-N-(7-methyl-5-phenyl-[1,2,4]triazolo[1,5-a]pyrimidin-2-yl)benzamide* (**13g**). The title compound was prepared starting from **13f** through Method B (4h) and purified by crystallization by EtOH, in 36% yield as light yellow crystals; mp 298-312 °C. ^1^H-NMR (DMSO-*d*_6_, 200 MHz): δ 2.75 (s, 3H, CH_3_), 6.75 (d, *J* = 8.9 Hz, 1H, aromatic CH), 7.30-7.40 (m, 2H, aromatic CH), 7.50–7.60 (m, 3H, aromatic CH), 7.80 (s, 1H, H-6), 8.15-8.25 (m, 2H, aromatic CH), 9.25 and 9.75 (s, each 1H, OH), 11.00 (s, 1H, NHCO).

##### General Procedure for the Preparation of 5,7-(substitued)-[1,2,4]triazolo[1,5-*a*]pyrimidin-2-amine (Method C)

A solution of 3,5-diamino-1,2,4-triazole (1.0 equiv.) and the appropriate β-diketone (1.0 equiv.) in acetic acid glacial was maintained at reflux until no starting material was detected by TLC (4–24 h). The reaction mixture was poured into ice/water affording a precipitate, which was purified as described below.

*5,7-Dimethyl-[1,2,4]triazolo[1,5-a]pyrimidin-2-amine* (**14**) [32]. The title compound was prepared by Method C (4 h) by using pentane-2,4-dione, and purified by treatment with Et_2_O, in 96% yield as light yellow solid; mp 179-202 °C. ^1^H-NMR (DMSO-*d*_6_, 200 MHz): δ 2.30 and 2.50 (s, each 3H, CH_3_), 6.20 (bs, 2H, NH_2_), 6.75 (s, 1H, H-6).

*4-Butyl-N-(5,7-dimethyl-[1,2,4]triazolo[1,5-a]pyrimidin-2-yl)benzamide* (**14b**). The title compound was prepared starting from **14** [32] through Method A (2 h) by using 4-butylbenzoyl chloride, and purified by flash chromatography eluting with DCM/MeOH (98:2), in 42% yield as white solid; mp 175–177 °C. ^1^H-NMR (DMSO-*d*_6_, 400 MHz): δ 0.90 (t, *J* = 7.0 Hz, 3H, CH_2_*CH_3_*), 1.25-1.35 (m, 2H, *CH_2_*CH_3_), 1.50-1.60 (m, 2H, CH_2_*CH_2_*CH_3_), 2.50 (s, 3H, CH_3_), 2.60–2.80 (m, 5H, C*CH_2_* and CH_3_), 7.00 (s, 1H, H-6), 7.25 (d, *J* = 7.1 Hz, 2H, aromatic CH), 7.95 (d, *J* = 7.1 Hz, 2H, aromatic CH), 11.10 (s, 1H, NHCO); ^13^C-NMR (DMSO-*d*_6_, 101 MHz): δ 14.1, 17.0, 22.1, 24.7, 33.1, 35.0, 110.8, 128.5, 128.6, 131.4, 146.9, 147.3, 154.1, 160.1, 164.2, 165.0.

*N-(5,7-Dimethyl-[1,2,4]triazolo[1,5-a]pyrimidin-2-yl)-2-fluorobenzamide* (**14c**). The title compound was prepared starting from **14** [32] through Method A (2 h) by using 2-fluorobenzoyl chloride, and purified by crystallization by EtOH, in 34% yield as white crystals; mp 195-200 °C. ^1^H-NMR (DMSO-*d*_6_, 400 MHz): δ 2.50 and 2.70 (s, each 3H, CH_3_), 7.10 (s, 1H, H-6), 7.25-7.35 (m, 2H, aromatic CH), 7.50–7.55 (m, 1H, aromatic CH), 7.65 (m, 1H, aromatic CH), 11.25 (s, 1H, NHCO).

*N-(5,7-Dimethyl-[1,2,4]triazolo[1,5-a]pyrimidin-2-yl)-4-nitrobenzamide* (**14d**). The title compound was prepared starting from **14** [32] through Method A (3 h) by using 4-nitrobenzoyl chloride, and purified by flash chromatography eluting with DCM/MeOH (98:2), in 63% yield as light yellow solid; mp 273–276 °C. ^1^H-NMR (DMSO-*d*_6_, 200 MHz): δ 2.50 and 2.70 (s, each 3H, CH_3_), 7.10 (s, 1H, H-6), 8.15 and 8.30 (d, *J* = 8.8 Hz, each 2H, aromatic CH), 11.70 (s, 1H, NHCO).

*4-Amino-N-(5,7-dimethyl-[1,2,4]triazolo[1,5-a]pyrimidin-2-yl)benzamide* (**14e**). The title compound was prepared starting from **14d** using the same procedure used for the synthesis of **12e** (3 h), and was purified by flash chromatography eluting with CHCl_3_/MeOH (98:2), in 74% yield as light yellow solid; mp 248-258 °C. ^1^H-NMR (DMSO-*d*_6_, 400 MHz): δ 2.50 and 2.70 (s, each 3H, CH_3_), 5.75 (s, 2H, NH_2_), 6.50 (d, *J* = 8.5 Hz, 2H, aromatic CH), 7.10 (s, 1H, H-6), 7.75 (d, *J* = 8.5 Hz, 2H, aromatic CH), 10.75 (s, 1H, NHCO).

*N-(5,7-Dimethyl-[1,2,4]triazolo[1,5-a]pyrimidin-2-yl)-3,4-dimethoxybenzamide* (**14f**). The title compound was prepared starting from **14** [32] through Method A (1 h) by using 3,4-dimethoxybenzoyl chloride, and purified by crystallization by cyclohexane/EtOAc mixture, in 38% yield as white crystals; mp 190-194 °C. ^1^H-NMR (DMSO-*d*_6_, 400 MHz): δ 2.50 and 2.70 (s, each 3H, CH_3_), 3.80–3.85 (m, 6H, OCH_3_), 7.05 (d, *J* = 8.4 Hz, each 1H, aromatic CH), 7.10 (s, 1H, H-6), 7.60-7.70 (m, 2H, aromatic CH), 11.10 (s, 1H, NHCO).

*N-(5,7-Dimethyl-[1,2,4]triazolo[1,5-a]pyrimidin-2-yl)-3,4-dihydroxybenzamide* (**14g**). The title compound was prepared starting from **14f** through Method B (16h) and purified by crystallization by EtOH, in 43% yield as white crystals; mp 299–301 °C. ^1^H-NMR (DMSO-*d*_6_, 400 MHz): δ 2.55 and 2.70 (s, each 3H, CH_3_), 6.80 (d, *J* = 7.9 Hz, 1H, aromatic CH), 7.20 (s, 1H, H-6), 7.45 (d, *J* = 7.8 Hz, 2H, aromatic CH), 11.10 (s, 1H, NHCO); ^13^C-NMR (DMSO-*d*_6_, 101 MHz): δ 17.06, 24.88, 111.34, 115.38, 116.32, 120.81, 124.80, 145.48, 147.24, 150.09, 153.47, 159.43, 164.76, 165.09. HRMS (ESI) *m/z* [M+H]+ calcd for C_14_H_13_N_5_O_3_ 300.10966, found 300.10964.

*5,7-Diphenyl-[1,2,4]triazolo[1,5-a]pyrimidin-2-amine* (**15**) [33]. The title compound was prepared by Method C (24h) by using 1,3-diphenylpropane-1,3-dione, and purified by flash chromatography eluting with CHCl_3_/MeOH (95:5), in 29% yield as light yellow solid; mp 198–199 °C. ^1^H-NMR (DMSO-*d*_6_, 200 MHz): δ 6.50 (bs, 2H, NH_2_), 7.40-7.60 (m, 6H, aromatic CH), 7.75 (s, 1H, H-6), 8.20-8.30 (m, 4H, aromatic CH).

*N-(5,7-diphenyl[1,2,4]triazolo[1,5-a]pyrimidin-2-yl)-3,4-dimethoxybenzamide* (**15f**). The title compound was prepared starting from **15** [33] through Method A (16h) by using 3,4-dimethoxybenzoyl chloride, and purified by flash chromatography eluting with cyclohexane: EtOAc (4:6), in 63% yield as white solid; mp 151–153 °C. ^1^H-NMR (DMSO-*d*_6_, 200 MHz) δ 3.80 and 3.85 (s, each 3H, OCH_3_), 7.10 (d, *J* = 8.4 Hz, 1H, aromatic CH), 7.55–7.75 (m, 8H, aromatic CH), 8.10 (s, 1H, H-6), 8.30-8.45 (m, 4H, aromatic CH), 11.10 (s, 1H, NHCO).

*N-(5,7-diphenyl-[1,2,4]triazolo[1,5-a]pyrimidin-2-yl)-3,4-dihydroxybenzamide* (**15g**). The title compound was prepared starting from **15f** through Method B (2 h) and purified by flash chromatography eluting with CHCl_3_/MeOH (99:1), in 32% yield as light brown solid; mp 258–260 °C. ^1^H-NMR (DMSO-*d*_6_, 400 MHz): δ 6.80 (d, *J* = 7.86 Hz, 1H, aromatic CH), 7.35–7.50 (m, 2H, aromatic CH), 7.50-7.70 (m, 6H, aromatic CH), 8.10 (s, 1H, H-6), 8.30–8.50 (m, 4H, aromatic CH), 9.50 (bs, 2H, OH), 11.00 (s, 1H, NHCO); ^13^C-NMR (DMSO-*d*_6_, 101 MHz): δ 106.5, 115.2, 116.2, 120.7, 124.8, 128.0, 128.9, 129.4, 130.1, 130.3, 131.5, 131.9, 136.6, 145.3, 147.0, 149.9, 155.4, 160.1, 161.3, 165.0. HRMS (ESI) *m/z* [M+H]+ calcd for C_24_H_17_N_5_O_3_ 424.14096, found 424.14119.

*7-(3,4-Dimethoxyphenyl)-5-methyl-[1,2,4]triazolo[1,5-a]pyrimidin-2-amine* (**16**). The title compound was prepared by Method C (3h) by using 1-(3,4-dimethoxyphenyl)-3-phenylpropane-1,3-dione [47], and purified by treatment with Et_2_O, in 95% yield as yellow solid. ^1^H-NMR (DMSO-*d*_6_, 400 MHz): δ 2.50 (s, 3H, CH_3_), 3.75 (s, 6H, OMe), 6.25 (bs, 2H, NH_2_), 7.05 (d, *J* = Hz, 1H, aromatic CH), 7.25 (s, 1H, aromatic CH), 7.75 (s, 1H, H-6), 7.90 (d, *J* = Hz 1H, aromatic CH).

*N-(7-(3,4-Dimethoxyphenyl)-5-methyl-[1,2,4]triazolo[1,5-a]pyrimidin-2-yl)benzamide* (**16h**) and *N-benzoyl-N-(7-(3,4-dimethoxyphenyl)-5-methyl-[1,2,4]triazolo[1,5-a]pyrimidin-2-yl)benzamide* (**16hh**). To the solution of **16** (0.40 g, 1.40 mmol) in dry DCM was added DIPEA (0.5 mL, 2.80 mmol) and 3,4-dimethoxybenzoyl chloride (0.39 g, 2.80 mmol). The reaction mixture was maintained at rt for 24 h. Then, it was poured into ice/water and extracted with DCM. The collected organic layers were dried over Na_2_SO_4_ and evaporated to dryness, affording a residue that was purified by flash chromatography eluting with CHCl_3_/MeOH (98:2), in 3% yield as a light yellow solid and 8% yield as white solid, respectively. ^1^H-NMR (DMSO-*d*_6_, 400 MHz) (**16h**): δ 2.60 (s, 3H, CH_3_), 3.80 and 3.85 (s, each 3 H, OCH_3_), 7.20 (d, *J* = 8.6 Hz, 1H, aromatic CH), 7.45 (t, *J* = 7.6 Hz, 2H, aromatic CH), 7.55-7.65 (m, 2H, aromatic CH), 7.90-8.00 (m, 3H, aromatic CH), 8.10 (d, *J* = 1.3 Hz, 1H, H-6), 11.40 (s, 1H, NHCO); (**16hh**): δ 2.60 (s, 3H, CH_3_), 3.70 and 3.80 (s, each 3H, OCH_3_), 7.10 (d, *J* = 8.6 Hz, 1H, aromatic CH), 7.40-7.50 (m, 6H, aromatic CH and H-6), 7.55-7.65 (m, 3H, aromatic CH), 7.80 (d, *J* = 7.4 Hz, 4H, aromatic CH); ^13^C-NMR (DMSO-*d*_6_, 101 MHz): δ 25.1, 56.0, 56.1, 110.6, 111.8, 112.5, 121.1, 123.4, 129.4, 129.5, 133.5, 133.8, 146.0, 148.8, 125.2, 155.7, 160.6, 166.3, 171.9. HRMS (ESI) *m*/*z* [M+Na]+ calcd for C_24_H_17_N_5_O_3_ 516.16477, found 516.16504.

*N-(7-(3,4-dihydroxyphenyl)-5-methyl-[1,2,4]triazolo[1,5-a]pyrimidin-2-yl)benzamide* (**17h**). The title compound was prepared starting from **16h** by Method B (overnight) and purified by crystallization by EtOH, in 71% yield as yellow crystals; mp 260-263 °C. ^1^H-NMR (DMSO-*d*_6_, 400 MHz): δ 2.60 (s, 3H, CH_3_), 6.95 (d, *J =* 8.4 Hz, 1H, aromatic CH), 7.35 (s, 1H, H-6), 7.50 (t, *J =* 7.5 Hz, 2H, aromatic CH), 7.62 (t, *J* = 7.4 Hz, 1H, aromatic CH), 7.69 (dd, *J* = 8.2, 2.2 Hz, 1H, aromatic CH), 7.75 (d, *J =* 2.1 Hz, 1H, aromatic CH), 8.00 (d, *J* = 7.5 Hz, 2H, aromatic CH), 9.35 and 9.90 (bs, each 1H, OH), 11.30 (s, 1H, NHCO); ^13^C-NMR (DMSO-*d*_6_, 101 MHz): δ 24.96, 108.91, 115.98, 117.28, 120.75, 122.43, 128.55 (2C), 128.91 (2C), 132.61, 134.06, 145.66, 146.32, 149.63, 155.46, 160.17, 164.56, 165.50. HRMS (ESI) *m/z* [M+H]+ calcd for C_19_H_15_N_5_O_3_ 362.12531, found 362.12522.

##### General Procedure for C-2 Amidation (Method D)

To a solution of the suitable carboxylic acid (1.0 equiv) in freshly distilled dry DCM was added oxalyl chloride (3 equiv) and after 30 min dry DMF (2 drops) was added. After 2 h, the reaction mixture was evaporated to dryness to give a residue that was dissolved in freshly distilled dry DCM and added to the appropriate aniline (1.0 equiv) and DIPEA (1.0 equiv). The reaction mixture was maintained at rt overnight. Then, reaction mixture was evaporated to dryness to give a residue that was poured with ice/water yielding a solid that was filtered and purified as described below.

*N-(3,4-Dimethoxyphenyl)-5-methyl-7-phenyl-[1,2,4]triazolo[1,5-a]pyrimidine-2-carboxamide* (**18f**). The title compound was prepared starting from **18** [31] by Method D (overnight) by using 3,4-dimethoxyaniline, and purified by flash chromatography eluting with CHCl_3_, in 26% yield as light pink solid. ^1^H-NMR (DMSO-*d*_6_, 400 MHz): δ 2.70 (s, 3H, CH_3_), 3.70 and 3.75 (s, each 3H, OCH_3_), 6.80 (d, *J* = 8.8 Hz, 1H, aromatic CH), 7.40 (dd, *J* = 2.2 and 8.7 Hz, 1H, aromatic CH), 7.55 (d, *J* = 2.1 Hz, 1H, H-6), 7.60–7.70 (m, 4H, aromatic CH), 8.15-8.25 (m, 2H, aromatic CH), 10.60 (s, 1H, NHCO).

*N-(3,4-Dihydroxyphenyl)-5-methyl-7-phenyl-[1,2,4]triazolo[1,5-a]pyrimidine-2-carboxamide* (**18g**). The title compound was prepared starting from **18f** by Method B (overnight) and purified by crystallization by EtOH, in 54% yield as yellow crystals; mp 278–281 °C. ^1^H-NMR (DMSO-*d*_6_, 400 MHz): δ 2.70 (s, 3H, CH_3_), 6.70 (d, *J* = 8.5 Hz, 1H, aromatic CH), 7.05 (dd, *J* = 8.5 and 2.0 Hz, 1H, aromatic CH), 7.40 (d, *J* = 2.1 Hz, 1H, H-6), 7.60-7.75 (m, 4H, aromatic CH), 8.15-8.25 (m, 2H, aromatic CH), 8.80 and 9.00 (s, each 1H, OH), 10.40 (s, 1H, NHCO); ^13^C-NMR (DMSO-*d*_6_, 101 MHz): δ 25.36, 109.61, 112.10, 112.44, 115.63, 129.22 (2C), 129.91, 130.07 (2C), 130.59, 132.17, 142.71, 145.33, 147.13, 155.94, 157.50, 159.64, 166.97. HRMS (ESI) *m/z* [M+H]+ calcd for C_19_H_15_N_5_O_3_ 362.12531, found 362.12491.

*N-(3,4-Dimethoxyphenyl)-7-methyl-5-phenyl-[1,2,4]triazolo[1,5-a]pyrimidine-2-carboxamide* (**19f**). The title compound was prepared starting from **19** [31] by Method D (overnight) by using 3,4-dimethoxyaniline, and purified by flash chromatography eluting with CHCl_3_, in 26% yield as brown solid. ^1^H-NMR (DMSO-*d*_6_, 200 MHz): δ 3.85 (s, 3H, CH_3_), 3.70–3.75 (s, each 3H, OCH_3_), 6.90 (d, *J* = 7.4, 1H, aromatic CH), 7.50-7.65 (m, 5H, aromatic CH), 8.10 (s, 1H, H-6), 8.25–8.35 (m, 2H, aromatic CH), 10.70 (s, 1H, NHCO).

*N-(3,4-Dihydroxyphenyl)-7-methyl-5-phenyl-[1,2,4]triazolo[1,5-a]pyrimidine-2-carboxamide* (**19g**) The title compound was prepared starting from **19f** by Method B (overnight) and purified by crystallization by EtOH, in 62% yield as brown crystals; mp 247–250 °C. ^1^H-NMR (DMSO-*d*_6_, 400 MHz): δ 2.90 (s, 3H, CH_3_), 6.70 (d, *J* = 8.4 Hz, 1H, aromatic CH), 7.10 (dd, *J* = 8.4 and 2.1 Hz, 1H, aromatic CH), 7.45 (d, *J* = 2.1 Hz, 1H, aromatic CH), 7.55-7.65 (m, 3H, aromatic CH), 8.10 (s, 1H, H-6), 8.25-8.35 (m, 2H, aromatic CH) 8.75 and 9.05 (s, each 1H, OH), 10.45 (s, 1H, NHCO); ^13^C-NMR (DMSO-*d*_6_, 101 MHz): δ 17.52, 108.94, 109.51, 112.36, 115.65, 128.10, 129.69, 130.69, 132.07, 136.35, 142.69, 145.32, 149.61, 155.25, 157.37, 160.01, 161.50. HRMS (ESI) *m/z* [M+H]+ calcd for C_19_H_15_N_5_O_3_ 362.12531, found 362.12485.

### 3.2. Biological Evaluation

#### 3.2.1. Expression and purification of recombinant HIV-1 RT

HIV-1 RT group M subtype B. Heterodimeric RT was expressed essentially as described [48]. Briefly, *E. coli* strain M15 containing the p6HRT-Prot vector was grown to an optical density at 600 nm of 0.7 and induced with 1.7 mM isopropyl β-D-1-thiogalactopyranoside for 4 h. Protein purification was carried out with a BioLogic LP system (Bio-Rad Laboratories, Hercules, CA, USA), using a combination of immobilized metal affinity and ion exchange chromatography. Cell pellets were re-suspended in lysis buffer (50 mM sodium phosphate buffer pH 7.8, containing 0.5 mg/mL lysozyme), incubated on ice for 20 min, and after adding NaCl to a final concentration of 0.3 M, were sonicated and centrifuged at 30,000×*g* for 1 h. The supernatant was loaded onto a Ni2+-NTA-Sepharose column pre-equilibrated with loading buffer (50 mM sodium phosphate buffer pH 7.8, containing 0.3M NaCl, 10% glycerol, and 10 mM imidazole) and washed thoroughly with wash buffer (50 mM sodium phosphate buffer pH 6.0, containing 0.3 M NaCl, 10% glycerol, and 80 mM imidazole). RT was eluted with an imidazole gradient in wash buffer (0–0.5). Fractions were collected, protein purity was checked by SDS-PAGE and found to exceed 90%. Enzyme-containing fractions were pooled and diluted 1:1 with 50 mM sodium phosphate buffer pH 7.0, containing 10% glycerol and then loaded into a Hi-trap heparin HP (GE Healthcare Lifescience, Little Chalfont, UK) pre-equilibrated with 10 column volumes of loading buffer (50 mM sodium phosphate buffer pH 7.0, containing 10% glycerol and 150 mM NaCl). The column was washed with loading buffer and the RT was eluted with Elute Buffer 2 (50 mM sodium phosphate pH 7.0, 10% glycerol, 1M NaCl). Fractions were collected, and purified protein was dialyzed and stored in buffer containing 50 mM Tris-HCl pH 7.0, 25 mM NaCl, 1 mM EDTA, and 50% glycerol. Catalytic activities and protein concentrations were determined. Enzyme-containing fractions were pooled and aliquots were stored at −80 °C.

#### 3.2.2. Site directed mutagenesis

Amino acid substitutions were introduced into the p66 HIV-1 RT subunit of a HIV-1 RT using a QuikChange mutagenesis kit, following the manufacturer’s instructions (Agilent Technologies Inc, Santa Clara, CA, USA).

#### 3.2.3. HIV-1 DNA polymerase-independent RNase H activity determination

HIV RT-associated RNase H activity was measured as described [49] in 100 µL reaction volume containing 50 mM Tris-HCl buffer pH 7.8, 6 mM MgCl_2_, 1 mM dithiothreitol (DTT), 80 mM KCl, 0.25 µM hybrid RNA/DNA 5′-GAUCUGAGCCUGGGAGCU-Fluorescin-3′ (HPLC, dry, QC: Mass Check) (available from Metabion International AG, Planegg/Steinkirchen, Germany) 5′-Dabcyl-AGCTCCCAGGCTCAGATC-3′ (HPLC, dry, QC: Mass Check) and 20 ng of wt RT or 700ng of Trp535Ala RT according to a linear range of dose-response curve. The reaction mixture was incubated for 10 min at 37 °C on a Victor 3 multilabel counter plate reader (model 1420-051, Perkin Elmer Waltham, MA, US) and product were quantified at 490/528 nm (excitation/emission wavelength).

#### 3.2.4. HIV-1 RNA-dependent DNA polymerase activity determination

RNA-dependent DNA polymerase (RDDP) activity was measured as described [50] in 25 µL volume containing 60 mM Tris-HCl buffer pH 8.1, 8 mM MgCl_2_, 60 mM KCl, 13 mM DTT, 2.5 µM poly(A)-oligo(dT),100 µM dTTP, and 6 ng of wt RT, according to a linear range of dose-response curve. After enzyme addition, the reaction mixture was incubated for 30 min at 37 °C and the stopped by addition of EDTA. Reaction products were detected by picogreen addition and measured with a Victor 3multilabel counter plate reader equipped with filters for 502/523 nm (excitation/emission wavelength).

#### 3.2.5. In Vitro Antiviral Assays

Evaluation of the antiviral activity of the compounds against HIV-1 strain III_B_ in MT-4 cells was performed using the MTT assay as previously described [51,52]. Stock solutions (10× the final concentration) of test compounds were added in 25 µL volumes to two series of triplicate wells so as to allow simultaneous evaluation of their effects on mock- and HIV-infected cells at the beginning of each experiment. Serial 5-fold dilutions of test compounds were made directly in flat-bottomed 96-well microtiter trays using a Biomek 3000 robot (Beckman Instruments, Fullerton, CA, USA). Untreated HIV- and mock-infected cell samples were included as controls. HIV-1(III_B_) stock (50 µl) at 100-300 CCID_50_ (50 % cell culture infectious doses) or culture medium was added to either the infected or mock-infected wells of the microtiter tray. Mock-infected cells were used to evaluate the effects of test compound on uninfected cells in order to assess the cytotoxicity of the test compounds. Exponentially growing MT-4 cells were centrifuged for 5 minutes at 220 g and the supernatant was discarded. The MT-4 cells were resuspended at 6 × 10^5^ cells/mL and 50 µl volumes were transferred to the microtiter tray wells. Five days after infection, the viability of mock-and HIV-infected cells was examined spectrophotometrically using the MTT assay. The MTT assay is based on the reduction of yellow colored 3-(4,5-dimethylthiazol-2-yl)-2,5-diphenyltetrazolium bromide (MTT) (Thermo Fisher Scientific, Waltham, MA, USA) by mitochondrial dehydrogenase activity in metabolically active cells to a blue-purple formazan that can be measured spectrophotometrically. The absorbances were read in an eight-channel computer-controlled photometer (Infinite M1000, Tecan, Männedorf, Switzerland), at two wavelengths (540 and 690 nm). All data were calculated using the median absorbance value of three wells. The 50% cytotoxic concentration (CC_50_) was defined as the concentration of the test compound that reduced the absorbance (OD_540_) of the mock-infected control sample by 50%. The concentration achieving 50% protection against the cytopathic effect of the virus in infected cells was defined as the 50% effective concentration (EC_50_).

### 3.3. Evaluation of MgCl_2_ Coordination

The coordination properties for the compounds was determined as reported [53]. Briefly, compounds were solubilized in 1 mL of 10% ethanol and 10 mM Tris–HCl, pH 7.8. The UV-Vis spectrum was recorded from 250 to 600 nm before and after addition of 6 mM MgCl_2_.

### 3.4. Evaluation of Fluorescence

The fluorescence of compounds in experimental conditions was verified in a black 96 multiwell plate in 100 µL volume of the RNase H reaction mixture 50 mM Tris-HCl buffer pH 7.8, 6 mM MgCl_2_, 1 mM DTT, 80 mM KCl, in absence (bik samples) and in presence of serial dilutions of compound. Samples with 0.25 µM hybrid RNA/DNA 5′-GAUCUGAGCCUGGGAGCU-fluorescin-3′, 5′-dabcyl-AGCTCCCAGGCTCAGATC-3′ were used with 20 ng of wt RT (fluorescein controls) and without enzyme (bik fluorescein mix). The plate was incubated at 37 °C for 1 h and fluorescence was measured on a Victor 3 multilabel counter plate reader at 490/528 nm (excitation/emission wavelengths).

### 3.5. In Silico Studies

#### 3.5.1. Selection of Crystal Structure Subset

From PDB the crystal structures data of HIV-1 RT were retrieved using the following query: “StructTitleQuery: struct.title.comparator=contains struct.title.value=HIV-1 REVERSE TRANSCRIPTASE and Resolution is between 0.0 and 2.5 and TAXONOMY is just Human immunodeficiency virus 1 and TAXONOMY is only just Human immunodeficiency virus 1”; accession date: 28/11/2019. From the results of this query, all the structures with mutated residues were discarded. According to RCSB PDB, we consider mutation only engineered mutations which are the genetic alteration deliberately introduced into a specific gene as opposed to spontaneously occurring genetic variation. Finally, 18 X-ray RT crystal structures were retained and downloaded.

#### 3.5.2. Binding Site Identification

The selected RT structures were submitted to the FTMap [38].Different probe clusters were identified by the software in the RNase H subunit. Hot spots within a distance of 5 Å were herein considered as hot spots composing the same pocket. For the pocket selection, we considered a distance of 20 Å from the Mg^2+^ ions of the RNase H catalytic site. For the druggability analysis, we used DogSiteScorer [41]. Pocket volume, calculated by counting the grid points constituting the pocket volume, DrugScore, a druggability estimation and SimpleScore, the linear combination of the three properties pocket volume, enclosure and lipophilic character, were the evaluated parameters.

#### 3.5.3. Molecular Docking Studies

Ligand-protein docking studies were performed using the Autodock 4.2.6 software [54]. The Graphical User Interface program “AutoDock Tools” was used to protein preparation and grid and docking parameter simulations. The Autodock atom type, and the charges were assigned, and polar hydrogens were added. Gasteiger charge was assigned and then non-polar hydrogens were merged. In the present study, the receptor grid of 60 × 60 × 60 was centered on the clusters’ centroid among the different crossclusters for the selected Site1. Prior to docking experiments, the designed compounds were built using the Schrodinger Maestro interface [55] and then submitted to the LigPrep utility [56] which rapidly produces low energy 3D structures considering ionization states, tautomers, stereochemistry, and ring conformations at the desired pH. For our study, a pH range of 7.5 ± 0.5 was set. All compounds were flexibly docked in a stepwise manner with using the Lamarckian Genetic Algorithm and empirical free energy, typically scoring functions. For each ligand, 100 docking runs was performed. The first three conformational clusters per ligand were considered and binding poses with a number in cluster (NiC) lower than 30 was discarded. The retained binding modes were thus considered reliable and were evaluated in terms of ligand binding energy (LBE) and NiC. All the reported figures were prepared by using Pymol 1.8 [57].

## 4. Conclusions

Despite the significant progress achieved so far in the treatment of HIV-1 infection, the identification of innovative agents able to exploit alternative targets or new binding sites on the traditional ones is still strongly required above all to counteract the burden of drug resistance. RT represents the most targeted HIV-encoded protein, with more than half of the approved anti-HIV-1 drugs inhibiting its associated polymerase function. Conversely, no inhibitor of the RT-associated RNase H activity progressed to clinic so far. However, over the past three decades, structurally different classes of RNHIs have been identified; some of them are dual inhibitors against both the RT-associated enzymatic functions, while others emerged as selective RNHIs.

Recently, by repurposing a series of anti-influenza cycloheptathiophene-3-carboxamide derivatives we enriched the class of allosteric RNHIs [27,28]. We here applied the same approach to another class of anti-influenza compounds developed by us identifying the TZP scaffold as a new chemotype suitable to achieve RNase H inhibition. Indeed, the structural investigation around the TZP core led to RNHIs, active in the sub-micromolar range. Spectrophotometric analysis showed that the TZP derivatives are unable to chelate divalent ions, thus indicating they are not active site RNHIs and suggesting an allosteric mechanism of action. As already highlighted for many allosteric RNHIs, the role of the catechol moiety in imparting anti-RNase H activity was pivotal also for the TZP class of derivatives. Nevertheless, we have ruled out a PAINS behaviour of our catechol-containing TZPs. The most potent 5-methyl-7-phenyl derivative **12g** and its C-2 inverse amide analogue **18g** showed anti-RNase H activity in the sub-micromolar range (IC_50_ = 0.8 and 0.41 µM, respectively), but this good activity did not result in anti-HIV-1 activity in cellular context. Other authors claimed for an ultrapotent RNase H inhibition in order to effectively compete with RNA:DNA duplex substrate thus achieving anti-HIV-1 activity [58].

Docking simulations located the putative binding site of TZPs into an allosteric pocket placed below the RNase H catalytic site, accessible from the back side of the RT protein, opposite to that one interested by the duplex binding. Within the proposed binding site, the occupation of the Hs1 by the C-7 aromatic ring represents an important feature for an efficient ligand binding. Of note, Hs1 seems big enough to host also bulkier substituents that could be exploited to improve the affinity. Interestingly, site-directed mutagenesis studies on residue Trp535, which is conserved among the HIV-1 infected patients [59], confirmed the docking results obtained for compound **18g**. The identification of this new allosteric binding pocket laid the foundation for future computational studies aimed at optimizing the TZP scaffold or identifying new chemotypes through virtual screening campaigns.

In conclusion, the discovery of allosteric RNHIs endowed with appropriate anti-HIV-1 activity as well as their optimization into leads for clinical assessment still remains a challenge. However, the RNase H represents a target definitely worth to be further explored. It could furnish an alternative therapeutic option not only against HIV infection but also to treat Hepatitis B virus (HBV) infection, which is another global health problem for whicH-News drugs are urgently needed [60].

## Figures and Tables

**Table 1:** Activity of TZP derivatives on the HIV-1 RT-associated RNase H and RDDP functions.

	Compound	R	HIV-1 RNaseH IC_50_, µM^a^	HIV-1 RDDP IC_50_, µM^b^
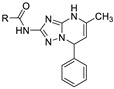	**11a**	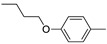	17.7 ± 2.7	>100 (74%)^c^
	**11b**	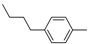	13.1 ± 2.2	>100 (74%)
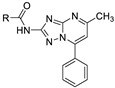	**12b**	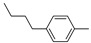	12.3 ± 1.5	>100 (59%)
	**12c**	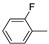	>100 (75%)	>100 (59%)
	**12d**	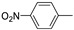	>100 (77%)	>100 (100%)
	**12e**	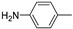	>100 (91%)	>100 (62%)
	**12f**	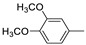	>100 (100%)	>100 (63%)
	**12g**	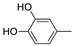	0.8 ± 0.2	>100 (73%)
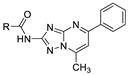	**13c**	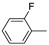	>100 (90%)	>100 (62%)
	**13d**	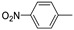	60.1 ± 3.3	>100 (72%)
	**13e**	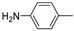	>100 (98%)	>100 (81%)
	**13f**	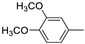	>100 (100%)	>100
	**13g**	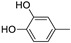	3.5 ± 1.5	>100
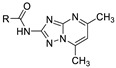	**14b**	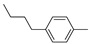	>100(100%)	
	**14c**	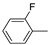	>100 (100%)	>100 (80%)
	**14d**	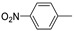	>100 (75%)	>100 (86%)
	**14e**	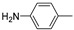	>100 (91%)	>100 (70%)
	**14f**	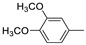	>100 (93%)	>100 (90%)
	**14g**	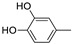	6.23 ± 0.01	>100 (78%)
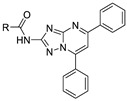	**15g**	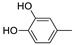	1.86 ± 0.54	20.5 ± 4.1
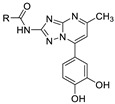	**17h**	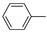	1.13 ± 0.33	>100 (60%)
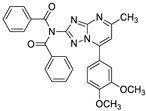	**16hh**	-	6.74 ± 0.55	>100 (55%)
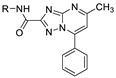	**18g**	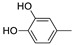	0.41 ± 0.005	>100 (67%)
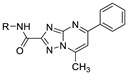	**19g**	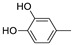	43.1 ± 2.1	N.T.^d^
	**5 (NSC727447)**	-	4.0 ± 0.5	N.T.
	**RDS1643**	-	9.0 ± 2.0	N.T.
	**efavirenz**	-	-	0.023 ± 0.0016

^a^ IC_50_: compound concentration required to inhibit the HIV-1 RT-associated RNase H activity by 50%. The table reports the average and standard deviation of three independent experiments, made in duplicate; ^b^ IC_50_: compound concentration required to inhibit the HIV-1 RT-associated RDDP activity by 50%. The table reports the average and standard deviation of three independent experiments, made in duplicate; ^c^: Percentage of residual enzymatic activity in the presence of 100 µM compound concentration; ^d^ N.T. = not tested.

## Supplementary Materials

The following are available online, Figure S1: Effect of MgCl_2_ on the spectrum of absorbance of compounds **12g**, **13g**, **14g**, **15g**, **16hh**, **17h**, and **18g**; Figure S2: 2D and 3D representation of **17h** binding mode produced by docking studies on 3LP1 structure; Figure S3: Effect of compounds **12g**, **13g**, **14g**, **15g**, **17h**, and **18g** on the fluorescein-based assay condition; Table S1: #Pprobes generated by FTmap for the analyzed crystal structures; Table S2: Predicted Ligand Binding Energy (LBE), predicted Ki (Ki_pred_) and number in cluster (NiC) for docked TZPs.
