# Supplementary material for: 1,2,4-Triazolo[1,5-a]pyrimidines as a Novel Class of Inhibitors of the HIV-1 Reverse Transcriptase-Associated Ribonuclease H Activity
Source: Molecules. 2020 Mar 5;25(5):1183. doi: 10.3390/molecules25051183 (PMC7179434; doi:10.3390/molecules25051183)
Supplement: Supplementary file 1 [file molecules-25-01183-s001.pdf]

molecules

Supplementary Materials

1,2,4-Triazolo[1,5-*a*]pyrimidines as a Novel Class of Inhibitors of the HIV-1 Reverse Transcriptase-Associated Ribonuclease H Activity

Jenny Desantis [1,2], Serena Massari [1], Angela Corona [3], Andrea Astolfi [1], Stefano Sabatini [1], Giuseppe Manfroni [1], Deborah Palazzotti [1], Violetta Cecchetti [1], Christophe Pannecouque [4], Enzo Tramontano [3] and Oriana Tabarrini [1,*]

[1] Department of Pharmaceutical Sciences, University of Perugia, 06123 Perugia, Italy; jenny.desantis@hotmail.it (J.D.); serena.massari@unipg.it (S.M.); andrea.astolfi@unipg.it (A.A.); stefano.sabatini@unipg.it (S.S.); giuseppe.manfroni@unipg.it (G.M.); deborah.palazzotti@studenti.unipg.it (D.P.); violetta.cecchetti@unipg.it (V.C.)

[2] Department of Chemistry, Biology, and Biotechnology, University of Perugia, Perugia, Italy

[3] Department of Life and Environmental Sciences, University of Cagliari, Cittadella Universitaria di Monserrato, Monserrato, 09042, Cagliari , Italy; angela.corona@unica.it (A.C.); tramon@unica.it (E.T.)

[4] Rega Institute for Medical Research, Laboratory of Virology and Chemotherapy, K.U. Leuven, 3000, Belgium; christophe.pannecouque@rega.kuleuven.be

* Correspondence: oriana.tabarrini@unipg.it; Tel.: +39-075-5855139

TABLE OF CONTENTS

Figure S1. Effect of $MgCl_2$ on the spectrum of absorbance of compounds **12g**, **13g**, **14g**, **15g**, **16hh**, **17h**, and **18g** S-2

Figure S2. 2D and 3D representation of **17h** binding mode produced by docking studies on 3LP1 structure S-4

Figure S3. Effect of compounds **12g**, **13g**, **14g**, **15g**, **18g**, and **17h** on the fluorescein-based assay condition S-5

Table S1. #Pprobes generated by FTmap for the analyzed crystal structures S-6

Table S2. Predicted Ligand Binding Energy (LBE), predicted Ki (Ki_{pred}) and number in cluster (NiC) for docked TZPs S-7

References S-7

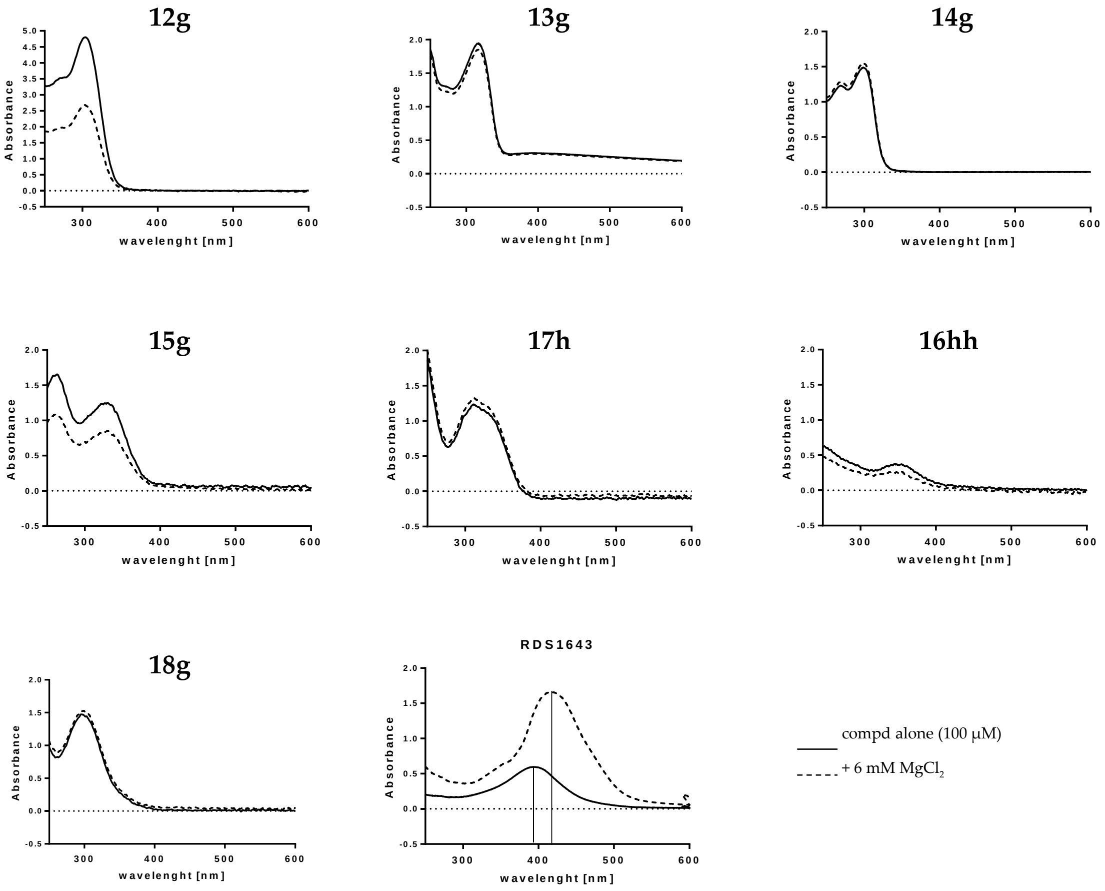

Figure S1. Effect of $MgCl_2$ on the spectrum of absorbance of compounds 12g, 13g, 14g, 15g, 16hh, 17h, and 18g. Mg^{2+} chelation UV/vis spectrum was measured with 100 μM of compound alone (unbroken line) or in the presence of 6mM $MgCl_2$ (dotted line). The active site RNHI RDS1643 [1] was used as reference compound.

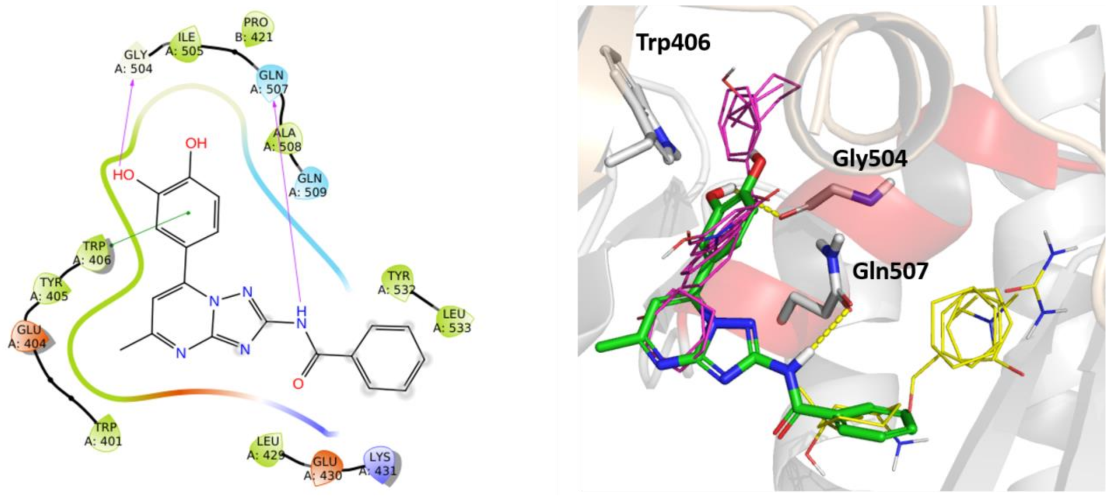

Figure S2. 2D and 3D representation of **17h** binding mode produced by docking studies on 3LP1 structure. Magenta lines, Hs1 probes; yellow lines, Hs2 probes; red cartoon, α-helix 14. Red cartoon, α-helix 14.

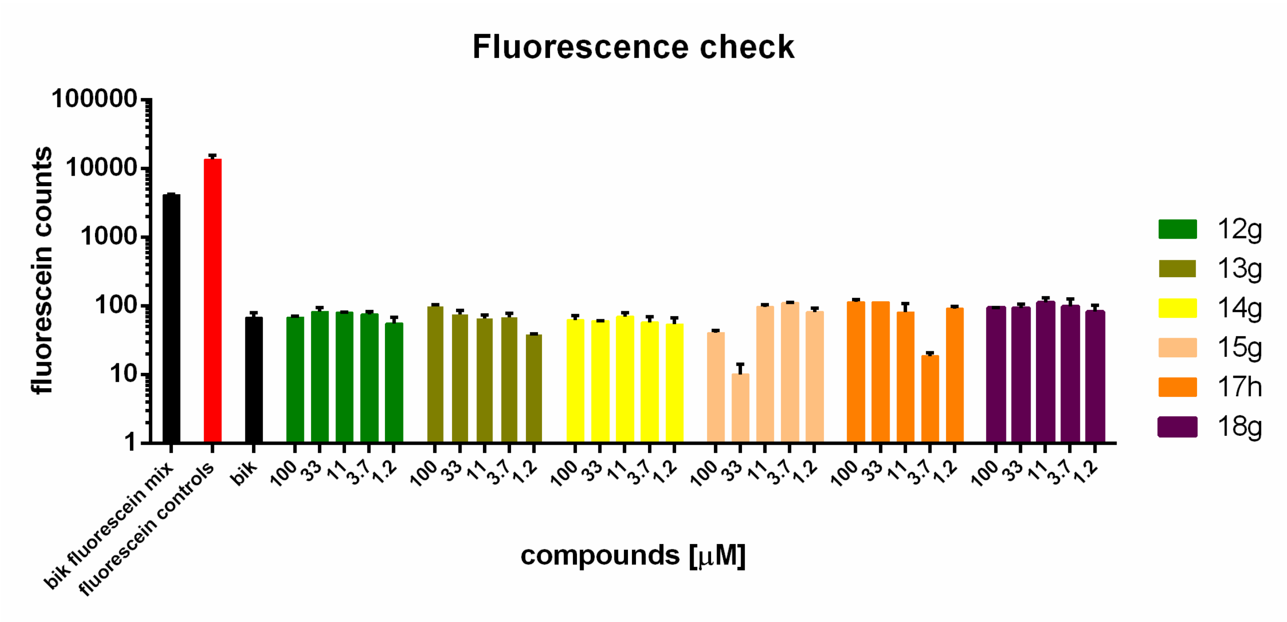

Figure S3. Effect of compounds **12g**, **13g**, **14g**, **15g**, **17h**, and **18g** on the fluorescein-based assay condition. Data are reported as the average and standard deviation of a triplicate.

Table S1. #Pprobes generated by FTMap for the analyzed crystal structures.

PDB ID	Site 1	Site 2	PDB Protein	Site 1	Site 2
**5K14**	20	/	**1C1B**	18	/
**2YNG**	9	15	**1C1C**	15	/
**2YNI**	10	6	**1EP4**	5	7
**3QIP**	15	/	**1FK9**	8	5
**3LP1**	20	/	**1JKH**	10	4
**3MEC**	7	/	**3DLE**	8	9
**3MEE**	19	10	**4I7F**	24	2
**3LAK**	11	11	**3DLG**	3	/
**1RTJ**	18	/	**2RKI**	16	6

Table S2. Predicted Ligand Binding Energy (LBE), predicted Ki (Ki_{pred}) and number in cluster (NiC) for docked TZPs.

**Compd**	**3LP1**			**4I7F**			**HIV-1 RNaseH IC_{50} (μM)**
	**LBE (Kcal/mol)**	**Ki_{pred} (μM)**	**NiC**	**LBE (Kcal/mol)**	**Ki_{pred} (μM)**	**NiC**	
**12g**	-9.07	0.23	58	-8.42	0.67	44	0.8
**13g**	-8.45	0.64	34	-8.54	0.55	84	3.5
**15g**	-9.39	0.13	61	-9.53	0.10	100	1.86
**17h**	-8.15	1.07	54	/	/	/	1.13
**18g**	-8.78	0.37	58	-9.02	0.25	68	0.41
**19g**	/	/	/	-8.25	0.89	65	43.1

References

1. Tramontano, E.; Esposito, F.; Badas, R.; Di Santo, R.; Costi, R.; La Colla, P. 6-[1-(4-Fluorophenyl)methyl-1*H*-pyrrol-2-yl)]-2,4-dioxo-5-hexenoic acid ethyl ester a novel diketo acid derivative which selectively inhibits the HIV-1 viral replication in cell culture and the ribonuclease H activity in vitro. *Antiviral Res.* **2005**, *65*, 117-124. doi: 10.1016/j.antiviral.2004.11.002
